# *syn*-Dispiro­[1,3-dioxolane-2,17′-penta­cyclo­[12.2.1.1^6,9^.0^2,13^.0^5,10^]octa­decane-18′,2′′-[1,3]dioxolane]-7′,15′-diene

**DOI:** 10.1107/S1600536810041565

**Published:** 2010-10-20

**Authors:** Rulla M. Kachlan, Macey C. Ruble, Jacob C. Timmerman, Markus Etzkorn, Daniel S. Jones

**Affiliations:** a Department of Chemistry, The University of North Carolina at Charlotte, 9201 University City Blvd., Charlotte, NC 28223, USA

## Abstract

The title compound, C_22_H_28_O_4_, is composed of a central octa­decane ring and two spiro­[bicyclo­[2.2.1]hept[2]ene-7,2′-[1,3]dioxolane] units. This polycycle has pseudo twofold symmetry and the central cyclo­octane ring has a distorted boat configuration.

## Related literature

For related structures, see: Garcia *et al.* (1991**a* ▶,b* ▶); Tenbusch *et al.* (2010 ▶). For the chemistry of *syn*-bis­quinoxalines, see: Chou *et al.* (2005 ▶); Etzkorn *et al.* (2010 ▶).
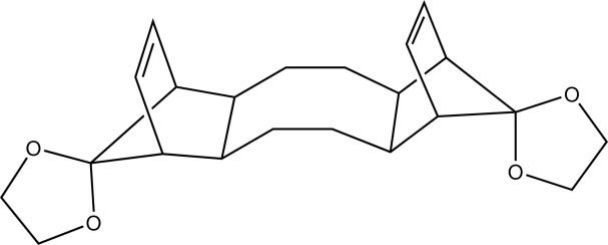


## Experimental

### 

#### Crystal data

C_22_H_28_O_4_
*M* *_r_* = 356.44
Monoclinic, 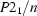

*a* = 11.4167 (11) Å
*b* = 6.7354 (7) Å
*c* = 24.185 (2) Å
β = 103.521 (9)°
*V* = 1808.2 (3) Å^3^
*Z* = 4
Cu *K*α radiation
μ = 0.71 mm^−1^
*T* = 295 K
0.35 × 0.20 × 0.20 mm

#### Data collection

Enraf–Nonius CAD-4 diffractometer
8422 measured reflections
3248 independent reflections
2693 reflections with *I* > 2σ(*I*)
*R*_int_ = 0.045
3 standard reflections every 79 reflections  intensity decay: 2%

#### Refinement

*R*[*F* ^2^ > 2σ(*F* ^2^)] = 0.039
*wR*(*F* ^2^) = 0.106
*S* = 1.05
3248 reflections
236 parameters
H-atom parameters constrained
Δρ_max_ = 0.24 e Å^−3^
Δρ_min_ = −0.19 e Å^−3^

### 

Data collection: *CAD-4 EXPRESS* (Enraf–Nonius, 1994 ▶); cell refinement: *CAD-4 EXPRESS*; data reduction: *XCAD4* (Harms & Wocadlo, 1995 ▶); program(s) used to solve structure: *SHELXS97* (Sheldrick, 2008 ▶); program(s) used to refine structure: *SHELXL97* (Sheldrick, 2008 ▶); molecular graphics: *ORTEP-3 for Windows* (Farrugia, 1997 ▶) and *Mercury* (Macrae, *et al.*, 2006 ▶); software used to prepare material for publication: *WinGX* (Farrugia, 1999 ▶).

## Supplementary Material

Crystal structure: contains datablocks global, I. DOI: 10.1107/S1600536810041565/su2208sup1.cif

Structure factors: contains datablocks I. DOI: 10.1107/S1600536810041565/su2208Isup2.hkl

Additional supplementary materials:  crystallographic information; 3D view; checkCIF report

## Figures and Tables

**Fig. 1.**
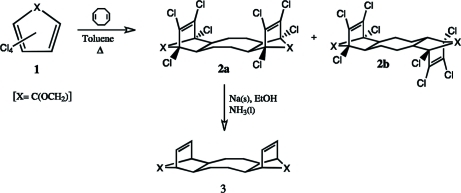
Synthesis scheme.

**Fig. 2.**
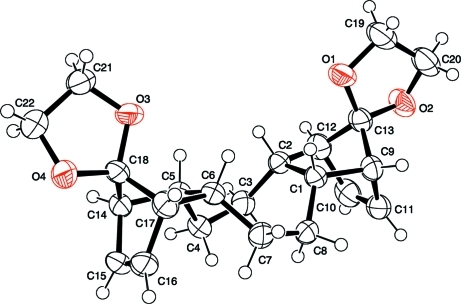
A view of the molecular structure of compound 3, with 50% probability displacement ellipsoids.

## supplementary crystallographic information

### Comment

The title compound is of interest as a non-chlorinated tether unit for *syn*-bisquinoxaline molecular tweezers. The non-chlorinated compounds are anticipated to display higher solubility in common organic solvents, thus facilitating the quantitative investigation of host–guest chemistry in solution. The title polycyclic molecule, 3, presented here was obtained by a twofold Diels-Alder reaction of cyclooctadiene and a cyclopentadieneone derivative, 1, followed by subsequent dehalogenation (Fig. 1). Larger molecular frameworks that incorporate scaffold 2a can be found in *syn*-bisquinoxalines that have previously been investigated for their luminescent properties (Chou *et al.*, 2005) and for their behavior as molecular tweezers (Etzkorn *et al.*, 2010). Compound 3 stems from the chlorinated derivative 2a, which was separated from its *anti*-isomer 2b*via* repeated recrystallization from diethyl ether, *i.e.*, the ether solution becomes more enriched in *syn*-isomer 2a*.* To improve the solubility of any molecular framework that is derived from scaffold 2a, we reduced the latter with sodium metal in ethanol and liquid ammonia to furnish 3 in good yield. The fully dechlorinated compound 3 did indeed show improved solubility in common organic solvents and, upon further functionalization to tweezer scaffolds, is expected to improve overall solubility.

The title molecule, 3, has pseudo 2-fold symmetry. The central cyclooctane has a distorted boat configuration (Fig. 2). The dioxalane ring (O1,C13,O2,C20,C19) has an envelope configuration with atom O2 at the flap, while ring (O3,C18,O4,C22,C21) has a half-chair configuration being twised about bond O4-C22.

A literature search revealed three related crystal structures. The first (Garcia *et al.*, 1991*a*) is similar to 3, but has the *anti*-orientation and an open ketal structure on each of the bridgehead carbon atoms. The second (Tenbusch *et al.*, 2010) is an octachloro derivative with the *anti*-orientation. The third (Garcia *et al.*, 1991**b*)* is an octachloro *syn*-structure with an open ketal arrangement on each of the bridgehead carbon atoms; this structure assumes the same distorted boat configuration as does compound 3.

### Experimental

The synthesis of the title compound, 3, is described in Fig. 1. A mixture of cycloocatdiene (3 g, 29 mmol) and spiroketal (1) (15 g, 57 mmol) was refluxed in toluene (3 ml) for three hours. The off-white paste was filtered, washed with methylene chloride (75 ml), dried, and washed again with cold methanol (15 ml) to remove traces of the mono-Diels-Alder adduct. The remaining colorless solid (14.5 g, 83%) was composed of a mixture of 2a and 2 b in a 1:4 ratio, respectively. After repeated recrystallization from diethyl ether, the pure *syn*-isomer 2a was obtained as colorless platelets (3.7 g, 21%).

A solution of 2a (1 g, 1.58 mmol) and THF (20 ml) was added to a mixture of liquid ammonia (250 ml) and ethanol (1.5 ml). Pieces of sodium metal (0.8 g, 29.6 mmol) were slowly added over two hours; the reaction mixture was then stirred for an additional hour. The reaction was quenched with solid ammonium chloride, and the ammonia was allowed to evaporate. The residue was taken up in water (75 ml) and the aqueous phase was extracted with methylene chloride (4 *x* 50 ml) to yield 3 as light-brown crystals (0.490 g). Purification of 3 by column chromatography (cyclohexane: ethyl acetate [4:1], *R*_f _= 0.11) afforded colorless crystals (0.439 g, 78%); *Mp*.>568 K. *IR (KBr)*: ~ν = 2955, 2856 (CH_2_), 1649 (C═C), 1473 (CH_2_ bend), 1303, 1290 (C—O—C), 1243, 1224, 1084, 1046, 819, 725 cm^-1^; *^1^**H NMR (CDCl**_3_**; 300 MHz):*δ = 6.18 (m, 4H, H-7',-8',-15',-16'), 3.98–3.90 (m, 4H, H-4,-4"), 3.85–3.76 (m, 4H, H-5,-5"), 2.86–2.72 (m, 4H, H-2',-5',-10'-13'), 2.46–2.4 (m, 4H, H-1',-6',-9',-13'); *^13^**C NMR (CDCl**_3_**; 75.6 MHz):*δ = 133.7 (C-7',-8',-15',-16'), 124.8 (C-17',-18'), 64.8 (C-4,-4"), 64.1 (C-5,-5"), 53.6 (C-2',-5',-10',-13'), 37.4 (C-1',-6', -9',-14'), 25.3 (C-3',-4',-11',-10').

### Refinement

The H-atoms were included in calculated positions and constrained using a riding model: C—H = 0.97 Å for methylene, 0.98 Å for methine, and 0.93 Å for olefinic H-atoms, with *U*_iso_(H) = 1.2U_eq_(C).

### Crystal data

**Table:**

C_22_H_28_O_4_	*F*(000) = 768
*M**_r_* = 356.44	*D*_x_ = 1.309 Mg m^−^^3^
Monoclinic, *P*2_1_/*n*	Cu *K*α radiation, λ = 1.54184 Å
Hall symbol: -P 2yn	Cell parameters from 25 reflections
*a* = 11.4167 (11) Å	θ = 15.3–42.6°
*b* = 6.7354 (7) Å	µ = 0.71 mm^−^^1^
*c* = 24.185 (2) Å	*T* = 295 K
β = 103.521 (9)°	Prism, colorless
*V* = 1808.2 (3) Å^3^	0.35 × 0.20 × 0.20 mm
*Z* = 4	

### Data collection

**Table:**

Enraf–Nonius CAD-4 diffractometer	θ_max_ = 67.5°, θ_min_ = 3.8°
Non–profiled ω/2θ scans	*h* = −13→0
8422 measured reflections	*k* = −8→8
3248 independent reflections	*l* = −28→28
2693 reflections with *I* > 2σ(*I*)	3 standard reflections every 79 reflections
*R*_int_ = 0.045	intensity decay: 2%

### Refinement

**Table:**

Refinement on *F*^2^	H-atom parameters constrained
Least-squares matrix: full	*w* = 1/[σ^2^(*F*_o_^2^) + (0.0414*P*)^2^ + 0.4578*P*] where *P* = (*F*_o_^2^ + 2*F*_c_^2^)/3
*R*[*F*^2^ > 2σ(*F*^2^)] = 0.039	(Δ/σ)_max_ < 0.001
*wR*(*F*^2^) = 0.106	Δρ_max_ = 0.24 e Å^−^^3^
*S* = 1.05	Δρ_min_ = −0.19 e Å^−^^3^
3248 reflections	Extinction correction: *SHELXL*, Fc^*^=kFc[1+0.001xFc^2^λ^3^/sin(2θ)]^-1/4^
236 parameters	Extinction coefficient: 0.0117 (6)
0 restraints	

### Special details

**Table:**

Geometry. Bond distances, angles etc. have been calculated using the rounded fractional coordinates. All su's are estimated from the variances of the (full) variance-covariance matrix. The cell esds are taken into account in the estimation of distances, angles and torsion angles

### Fractional atomic coordinates and isotropic or equivalent isotropic displacement parameters (Å^2^)

**Table:**

	*x*	*y*	*z*	*U*_iso_*/*U*_eq_	
O1	0.49270 (9)	0.9050 (2)	−0.18532 (5)	0.0544 (4)	
O2	0.56491 (10)	1.0106 (2)	−0.25975 (4)	0.0545 (4)	
O3	0.58431 (10)	0.6331 (2)	0.05326 (5)	0.0597 (4)	
O4	0.68575 (10)	0.66022 (19)	0.14523 (4)	0.0534 (4)	
C1	0.68153 (12)	1.0631 (2)	−0.10437 (6)	0.0351 (4)	
C2	0.72139 (12)	0.8459 (2)	−0.11521 (6)	0.0360 (4)	
C3	0.84705 (13)	0.7735 (2)	−0.08405 (6)	0.0419 (5)	
C4	0.87904 (12)	0.7689 (2)	−0.01860 (6)	0.0413 (5)	
C5	0.77291 (12)	0.7502 (2)	0.00928 (6)	0.0348 (4)	
C6	0.70386 (12)	0.9466 (2)	0.01680 (6)	0.0362 (4)	
C7	0.75302 (14)	1.1468 (2)	0.00213 (6)	0.0423 (5)	
C8	0.76760 (13)	1.1811 (2)	−0.05857 (6)	0.0399 (5)	
C9	0.65659 (13)	1.1569 (2)	−0.16468 (6)	0.0416 (5)	
C10	0.80267 (14)	0.9816 (3)	−0.19355 (7)	0.0535 (6)	
C11	0.77312 (14)	1.1653 (3)	−0.18360 (6)	0.0509 (6)	
C12	0.70828 (13)	0.8439 (3)	−0.18100 (6)	0.0445 (5)	
C13	0.59793 (13)	0.9774 (3)	−0.20041 (6)	0.0430 (5)	
C14	0.80596 (13)	0.6639 (2)	0.07062 (6)	0.0408 (5)	
C15	0.89060 (14)	0.8058 (3)	0.10824 (6)	0.0484 (5)	
C16	0.82908 (15)	0.9654 (3)	0.11512 (6)	0.0500 (5)	
C17	0.70117 (14)	0.9382 (2)	0.08140 (6)	0.0428 (5)	
C18	0.68912 (13)	0.7153 (2)	0.08916 (6)	0.0419 (5)	
C19	0.39160 (16)	0.9459 (4)	−0.23040 (8)	0.0676 (7)	
C20	0.44134 (16)	1.0615 (3)	−0.27232 (8)	0.0645 (7)	
C21	0.53301 (14)	0.4946 (3)	0.08401 (7)	0.0488 (5)	
C22	0.62046 (16)	0.4807 (3)	0.14092 (8)	0.0564 (6)	
H1	0.60440	1.05420	−0.09330	0.0420*	
H2	0.66180	0.75390	−0.10640	0.0430*	
H3A	0.90590	0.85690	−0.09600	0.0500*	
H3B	0.85730	0.64010	−0.09730	0.0500*	
H4A	0.93320	0.65830	−0.00620	0.0500*	
H4B	0.92250	0.88970	−0.00490	0.0500*	
H5	0.71460	0.65960	−0.01410	0.0420*	
H6	0.62090	0.93370	−0.00570	0.0430*	
H7C	0.70030	1.24980	0.01050	0.0510*	
H7D	0.83120	1.16620	0.02780	0.0510*	
H8C	0.75610	1.32130	−0.06740	0.0480*	
H8D	0.84950	1.14760	−0.05990	0.0480*	
H9	0.60900	1.27950	−0.16970	0.0500*	
H10	0.87030	0.94370	−0.20620	0.0640*	
H11	0.81640	1.27930	−0.18760	0.0610*	
H12	0.70340	0.71250	−0.19880	0.0530*	
H14	0.82980	0.52380	0.07380	0.0490*	
H15	0.97210	0.78450	0.12390	0.0580*	
H16	0.85920	1.07580	0.13700	0.0600*	
H17	0.64040	1.02140	0.09280	0.0510*	
H19G	0.33210	1.02310	−0.21700	0.0810*	
H19H	0.35450	0.82380	−0.24720	0.0810*	
H20E	0.40210	1.02430	−0.31100	0.0770*	
H20F	0.43090	1.20290	−0.26760	0.0770*	
H21A	0.52350	0.36670	0.06510	0.0590*	
H21B	0.45480	0.53980	0.08820	0.0590*	
H22C	0.57840	0.46940	0.17130	0.0680*	
H22D	0.67340	0.36730	0.14240	0.0680*	

### Atomic displacement parameters (Å^2^)

**Table:**

	*U*^11^	*U*^22^	*U*^33^	*U*^12^	*U*^13^	*U*^23^
O1	0.0405 (6)	0.0779 (9)	0.0398 (6)	−0.0133 (5)	−0.0009 (4)	0.0090 (6)
O2	0.0556 (6)	0.0750 (9)	0.0288 (5)	0.0006 (6)	0.0017 (5)	0.0037 (5)
O3	0.0506 (6)	0.0824 (9)	0.0389 (6)	−0.0342 (6)	−0.0039 (5)	0.0101 (6)
O4	0.0637 (7)	0.0621 (8)	0.0314 (6)	−0.0194 (6)	0.0048 (5)	0.0056 (5)
C1	0.0343 (7)	0.0386 (8)	0.0312 (7)	−0.0020 (6)	0.0055 (5)	0.0016 (6)
C2	0.0367 (7)	0.0384 (8)	0.0319 (7)	−0.0039 (6)	0.0061 (5)	0.0006 (6)
C3	0.0398 (7)	0.0443 (9)	0.0424 (8)	0.0042 (6)	0.0113 (6)	0.0030 (7)
C4	0.0342 (7)	0.0452 (9)	0.0412 (8)	0.0014 (6)	0.0021 (6)	0.0035 (7)
C5	0.0355 (7)	0.0340 (8)	0.0311 (7)	−0.0065 (6)	0.0000 (5)	−0.0010 (6)
C6	0.0367 (7)	0.0375 (8)	0.0315 (7)	−0.0043 (6)	0.0021 (5)	−0.0010 (6)
C7	0.0535 (8)	0.0346 (8)	0.0363 (8)	−0.0053 (6)	0.0055 (6)	−0.0029 (6)
C8	0.0460 (8)	0.0331 (8)	0.0382 (8)	−0.0057 (6)	0.0051 (6)	0.0018 (6)
C9	0.0464 (8)	0.0421 (9)	0.0339 (8)	0.0000 (6)	0.0046 (6)	0.0049 (7)
C10	0.0456 (8)	0.0806 (13)	0.0365 (8)	0.0042 (8)	0.0143 (7)	0.0091 (9)
C11	0.0487 (9)	0.0662 (12)	0.0355 (8)	−0.0129 (8)	0.0052 (7)	0.0122 (8)
C12	0.0518 (9)	0.0470 (10)	0.0338 (8)	0.0015 (7)	0.0084 (6)	−0.0043 (7)
C13	0.0442 (8)	0.0543 (10)	0.0283 (7)	−0.0047 (7)	0.0041 (6)	0.0033 (7)
C14	0.0444 (8)	0.0380 (8)	0.0346 (8)	−0.0049 (6)	−0.0017 (6)	0.0030 (6)
C15	0.0410 (8)	0.0607 (11)	0.0358 (8)	−0.0132 (7)	−0.0063 (6)	0.0049 (8)
C16	0.0623 (10)	0.0507 (10)	0.0313 (8)	−0.0204 (8)	−0.0006 (7)	−0.0055 (7)
C17	0.0493 (8)	0.0454 (9)	0.0328 (8)	−0.0034 (7)	0.0079 (6)	−0.0024 (7)
C18	0.0427 (8)	0.0517 (9)	0.0265 (7)	−0.0136 (7)	−0.0017 (6)	0.0020 (7)
C19	0.0475 (9)	0.0934 (16)	0.0524 (11)	−0.0054 (10)	−0.0072 (8)	0.0120 (10)
C20	0.0559 (10)	0.0827 (15)	0.0455 (10)	0.0009 (9)	−0.0074 (8)	0.0076 (10)
C21	0.0448 (8)	0.0518 (10)	0.0501 (9)	−0.0117 (7)	0.0118 (7)	0.0008 (8)
C22	0.0569 (10)	0.0599 (11)	0.0510 (10)	−0.0133 (8)	0.0099 (8)	0.0112 (9)

### Geometric parameters (Å, °)

**Table:**

O1—C13	1.4210 (19)	C19—C20	1.492 (3)
O1—C19	1.417 (2)	C21—C22	1.503 (3)
O2—C13	1.4138 (17)	C1—H1	0.9800
O2—C20	1.414 (2)	C2—H2	0.9800
O3—C18	1.4165 (19)	C3—H3A	0.9700
O3—C21	1.404 (2)	C3—H3B	0.9700
O4—C18	1.4151 (17)	C4—H4A	0.9700
O4—C22	1.411 (2)	C4—H4B	0.9700
C1—C2	1.5722 (19)	C5—H5	0.9800
C1—C8	1.522 (2)	C6—H6	0.9800
C1—C9	1.554 (2)	C7—H7C	0.9700
C2—C3	1.536 (2)	C7—H7D	0.9700
C2—C12	1.563 (2)	C8—H8C	0.9700
C3—C4	1.539 (2)	C8—H8D	0.9700
C4—C5	1.523 (2)	C9—H9	0.9800
C5—C6	1.5721 (19)	C10—H10	0.9300
C5—C14	1.555 (2)	C11—H11	0.9300
C6—C7	1.534 (2)	C12—H12	0.9800
C6—C17	1.571 (2)	C14—H14	0.9800
C7—C8	1.533 (2)	C15—H15	0.9300
C9—C11	1.506 (2)	C16—H16	0.9300
C9—C13	1.545 (2)	C17—H17	0.9800
C10—C11	1.319 (3)	C19—H19G	0.9700
C10—C12	1.506 (2)	C19—H19H	0.9700
C12—C13	1.530 (2)	C20—H20E	0.9700
C14—C15	1.505 (2)	C20—H20F	0.9700
C14—C18	1.543 (2)	C21—H21A	0.9700
C15—C16	1.316 (3)	C21—H21B	0.9700
C16—C17	1.508 (2)	C22—H22C	0.9700
C17—C18	1.5232 (19)	C22—H22D	0.9700
			
C13—O1—C19	108.73 (13)	H3A—C3—H3B	107.00
C13—O2—C20	105.81 (12)	C3—C4—H4A	108.00
C18—O3—C21	109.41 (12)	C3—C4—H4B	108.00
C18—O4—C22	106.62 (12)	C5—C4—H4A	108.00
C2—C1—C8	116.40 (12)	C5—C4—H4B	108.00
C2—C1—C9	102.62 (11)	H4A—C4—H4B	107.00
C8—C1—C9	114.61 (11)	C4—C5—H5	107.00
C1—C2—C3	119.16 (11)	C6—C5—H5	107.00
C1—C2—C12	102.43 (12)	C14—C5—H5	107.00
C3—C2—C12	110.67 (12)	C5—C6—H6	108.00
C2—C3—C4	118.74 (12)	C7—C6—H6	108.00
C3—C4—C5	115.77 (12)	C17—C6—H6	108.00
C4—C5—C6	116.99 (11)	C6—C7—H7C	108.00
C4—C5—C14	114.35 (12)	C6—C7—H7D	108.00
C6—C5—C14	102.74 (11)	C8—C7—H7C	108.00
C5—C6—C7	119.51 (12)	C8—C7—H7D	108.00
C5—C6—C17	102.22 (11)	H7C—C7—H7D	107.00
C7—C6—C17	110.80 (11)	C1—C8—H8C	108.00
C6—C7—C8	118.79 (12)	C1—C8—H8D	109.00
C1—C8—C7	114.97 (12)	C7—C8—H8C	109.00
C1—C9—C11	108.64 (12)	C7—C8—H8D	109.00
C1—C9—C13	99.63 (11)	H8C—C8—H8D	108.00
C11—C9—C13	99.07 (12)	C1—C9—H9	116.00
C11—C10—C12	108.38 (15)	C11—C9—H9	116.00
C9—C11—C10	107.58 (15)	C13—C9—H9	116.00
C2—C12—C10	107.29 (13)	C11—C10—H10	126.00
C2—C12—C13	100.56 (12)	C12—C10—H10	126.00
C10—C12—C13	98.80 (15)	C9—C11—H11	126.00
O1—C13—O2	105.91 (12)	C10—C11—H11	126.00
O1—C13—C9	114.01 (13)	C2—C12—H12	116.00
O1—C13—C12	113.87 (15)	C10—C12—H12	116.00
O2—C13—C9	114.95 (15)	C13—C12—H12	116.00
O2—C13—C12	114.18 (13)	C5—C14—H14	116.00
C9—C13—C12	94.02 (12)	C15—C14—H14	116.00
C5—C14—C15	108.52 (12)	C18—C14—H14	116.00
C5—C14—C18	99.20 (11)	C14—C15—H15	126.00
C15—C14—C18	99.11 (11)	C16—C15—H15	126.00
C14—C15—C16	108.01 (14)	C15—C16—H16	126.00
C15—C16—C17	108.08 (15)	C17—C16—H16	126.00
C6—C17—C16	106.97 (12)	C6—C17—H17	116.00
C6—C17—C18	100.45 (11)	C16—C17—H17	116.00
C16—C17—C18	99.04 (13)	C18—C17—H17	116.00
O3—C18—O4	106.06 (12)	O1—C19—H19G	111.00
O3—C18—C14	113.46 (12)	O1—C19—H19H	111.00
O3—C18—C17	113.42 (12)	C20—C19—H19G	111.00
O4—C18—C14	116.03 (12)	C20—C19—H19H	111.00
O4—C18—C17	113.58 (12)	H19G—C19—H19H	109.00
C14—C18—C17	94.37 (11)	O2—C20—H20E	111.00
O1—C19—C20	104.67 (15)	O2—C20—H20F	111.00
O2—C20—C19	104.27 (15)	C19—C20—H20E	111.00
O3—C21—C22	104.87 (14)	C19—C20—H20F	111.00
O4—C22—C21	103.92 (15)	H20E—C20—H20F	109.00
C2—C1—H1	108.00	O3—C21—H21A	111.00
C8—C1—H1	108.00	O3—C21—H21B	111.00
C9—C1—H1	108.00	C22—C21—H21A	111.00
C1—C2—H2	108.00	C22—C21—H21B	111.00
C3—C2—H2	108.00	H21A—C21—H21B	109.00
C12—C2—H2	108.00	O4—C22—H22C	111.00
C2—C3—H3A	108.00	O4—C22—H22D	111.00
C2—C3—H3B	108.00	C21—C22—H22C	111.00
C4—C3—H3A	108.00	C21—C22—H22D	111.00
C4—C3—H3B	108.00	H22C—C22—H22D	109.00
			
C19—O1—C13—O2	−16.0 (2)	C5—C6—C7—C8	59.18 (18)
C19—O1—C13—C9	111.44 (16)	C17—C6—C7—C8	177.55 (13)
C19—O1—C13—C12	−142.24 (16)	C5—C6—C17—C16	68.32 (14)
C13—O1—C19—C20	−4.0 (2)	C5—C6—C17—C18	−34.58 (14)
C20—O2—C13—O1	30.52 (18)	C7—C6—C17—C16	−60.10 (16)
C20—O2—C13—C9	−96.31 (16)	C7—C6—C17—C18	−162.99 (12)
C20—O2—C13—C12	156.61 (15)	C6—C7—C8—C1	27.76 (19)
C13—O2—C20—C19	−32.55 (19)	C1—C9—C11—C10	70.25 (15)
C21—O3—C18—O4	−13.28 (16)	C13—C9—C11—C10	−33.20 (15)
C21—O3—C18—C14	115.23 (14)	C1—C9—C13—O1	58.67 (16)
C21—O3—C18—C17	−138.60 (14)	C1—C9—C13—O2	−178.77 (12)
C18—O3—C21—C22	−5.34 (18)	C1—C9—C13—C12	−59.72 (12)
C22—O4—C18—O3	27.73 (16)	C11—C9—C13—O1	169.48 (13)
C22—O4—C18—C14	−99.25 (15)	C11—C9—C13—O2	−67.95 (15)
C22—O4—C18—C17	152.96 (14)	C11—C9—C13—C12	51.10 (13)
C18—O4—C22—C21	−30.43 (17)	C12—C10—C11—C9	−0.71 (17)
C8—C1—C2—C3	−5.80 (18)	C11—C10—C12—C2	−69.33 (17)
C8—C1—C2—C12	−128.27 (13)	C11—C10—C12—C13	34.70 (16)
C9—C1—C2—C3	120.19 (13)	C2—C12—C13—O1	−60.17 (16)
C9—C1—C2—C12	−2.28 (14)	C2—C12—C13—O2	178.01 (14)
C2—C1—C8—C7	−85.21 (15)	C2—C12—C13—C9	58.33 (13)
C9—C1—C8—C7	155.07 (12)	C10—C12—C13—O1	−169.73 (13)
C2—C1—C9—C11	−64.62 (14)	C10—C12—C13—O2	68.45 (17)
C2—C1—C9—C13	38.44 (13)	C10—C12—C13—C9	−51.23 (13)
C8—C1—C9—C11	62.51 (16)	C5—C14—C15—C16	70.53 (16)
C8—C1—C9—C13	165.58 (12)	C18—C14—C15—C16	−32.44 (15)
C1—C2—C3—C4	60.36 (17)	C5—C14—C18—O3	57.69 (14)
C12—C2—C3—C4	178.65 (13)	C5—C14—C18—O4	−179.12 (11)
C1—C2—C12—C10	67.63 (15)	C5—C14—C18—C17	−60.19 (12)
C1—C2—C12—C13	−35.14 (15)	C15—C14—C18—O3	168.29 (12)
C3—C2—C12—C10	−60.42 (17)	C15—C14—C18—O4	−68.52 (15)
C3—C2—C12—C13	−163.20 (12)	C15—C14—C18—C17	50.41 (12)
C2—C3—C4—C5	25.27 (17)	C14—C15—C16—C17	−1.07 (17)
C3—C4—C5—C6	−82.83 (15)	C15—C16—C17—C6	−69.25 (16)
C3—C4—C5—C14	157.02 (11)	C15—C16—C17—C18	34.67 (16)
C4—C5—C6—C7	−6.45 (18)	C6—C17—C18—O3	−59.54 (15)
C4—C5—C6—C17	−129.14 (13)	C6—C17—C18—O4	179.28 (12)
C14—C5—C6—C7	119.68 (13)	C6—C17—C18—C14	58.38 (12)
C14—C5—C6—C17	−3.01 (13)	C16—C17—C18—O3	−168.80 (12)
C4—C5—C14—C15	63.85 (15)	C16—C17—C18—O4	70.02 (15)
C4—C5—C14—C18	166.77 (11)	C16—C17—C18—C14	−50.88 (12)
C6—C5—C14—C15	−63.96 (14)	O1—C19—C20—O2	22.4 (2)
C6—C5—C14—C18	38.95 (12)	O3—C21—C22—O4	21.88 (18)
